# Draft Genome Sequence of Nitrosococcus oceani Strain NS58, a Marine Ammonia-Oxidizing Gammaproteobacterium Isolated from Tokyo Bay Sediment

**DOI:** 10.1128/MRA.00923-19

**Published:** 2019-08-29

**Authors:** Hideo Dohra, Kentaro Arai, Hidetoshi Urakawa, Taketomo Fujiwara

**Affiliations:** a Instrumental Research Support Office, Research Institute of Green Science and Technology, Shizuoka University, Shizuoka, Japan; b Department of Biological Science, Graduate School of Science, Shizuoka University, Shizuoka, Japan; c Department of Marine and Ecological Sciences, Florida Gulf Coast University, Fort Myers, Florida, USA; d Department of Environment and Energy Systems, Graduate School of Science and Technology, Shizuoka University, Shizuoka, Japan; Georgia Institute of Technology

## Abstract

We report a draft genome sequence of Nitrosococcus oceani strain NS58, isolated from Tokyo Bay sediment. The genome sequence of strain NS58 was nearly identical (>99.99%) to those of other strains of *N. oceani* isolated from different ocean regions. Only nine single-nucleotide polymorphisms were identified between *N. oceani* ATCC 19707^T^ and NS58.

## ANNOUNCEMENT

Nitrosococcus oceani, formerly designated *Nitrosocystis oceanus*, is a cosmopolitan marine gammaproteobacterial ammonia oxidizer found in various ocean regions (1, 2). Years after the first report of this species in 1962, the first genome sequence was determined in 2006 (2, 3). We isolated an ammonia-oxidizing bacterium designated strain NS58 from Tokyo Bay sediment by using a combination of serial dilution and gellan gum plating techniques (4, 5). The isolate was identified as *N. oceani* based on 16S rRNA phylogenetic analysis (6). Electron microscopic observation of the isolate demonstrated the presence of a thylakoid-like intracellular structure in the oval-shaped cells, which is a characteristic of *N. oceani* (6, 7). Strain NS58 has been characterized by its biochemical features of hydroxylamine oxidation, nitrifier denitrification, and nitrous oxide generation (6, 8, 9).

Strain NS58 was cultivated aerobically in an (NH_4_)_2_SO_4_-supplemented artificial seawater medium at 25°C in the dark (6). Genomic DNA of *N. oceani* strain NS58 was extracted using the DNeasy blood and tissue kit (Qiagen) and fragmented using the Covaris acoustic solubilizer, as previously reported (10). The library constructed using the TruSeq DNA PCR-free library prep kit (Illumina) was sequenced using the Illumina MiSeq platform (301-bp paired-end reads). Adapter sequences and low-quality ends (quality score, <15) of the raw reads were trimmed using Trimmomatic ver. 0.36 (11). The resultant 799,711 high-quality read pairs, totaling 428 Mb and representing 122-fold coverage of the genome, were assembled using SPAdes ver. 3.13.0 (12) with a combination of the default set of *k*-mer sizes and options (–careful, –only-assembler, and –cov-cutoff auto), as previously described (10). The draft genome consisted of 54 contigs (>200 bp) totaling 3,508,376 bp with a G+C content of 50.33% and included one plasmid (designated pNS58) identical to the plasmid A (GenBank accession number CP000126) of ATCC 19707^T^. The genome was annotated using DFAST-core ver. 1.2.0 (13), using all protein sequences of *N. oceani* ATCC 19707^T^ as references. The genome contained 3,162 protein-coding sequences, 6 rRNA genes, and 45 tRNA genes.

Average nucleotide identity (ANI) analysis (14, 15) with ATCC 19707^T^ (3) and three other *N. oceani* strains whose genome sequences have been released (1) demonstrated that the NS58 genome showed extremely high ANI values (>99.99%) with ATCC 19707^T^, C-27, and AFC27 and a moderate ANI value (98.28%) with AFC132 (Table 1). Further, we analyzed single-nucleotide polymorphisms (SNPs) and short insertions/deletions in the NS58 genome compared to ATCC 19707^T^ as a reference. High-quality reads were aligned to the ATCC 19707^T^ genome (GenBank accession numbers CP000126 and CP000127) using the Burrows-Wheeler Aligner MEM algorithm (BWA-MEM) ver. 0.7.12 (16). SNPs were called using HaplotypeCaller in the Genome Analysis Toolkit ver. 3.7 (17), and their functional effects were predicted using SnpEff ver. 4.3T (18). As a result, only nine SNPs were identified in the NS58 genome. The SNPs were not found in genes encoding ammonia monooxygenase (*amoABC*), hydroxylamine oxidoreductase gene clusters, or *nirK* and *norB* genes that participate in nitrifier denitrification in NS58. Rather, the nucleotide sequences of these genes were identical to the counterparts in ATCC 19707^T^. Thus, the genomes of four of the five sequenced strains of *N. oceani*, ATCC 19707^T^, C-27, AFC27, and NS58, were nearly identical despite being isolated from different ocean regions (Table 1). This genetic homogeneity of *N. oceani*, a widely distributed ammonia-oxidizing marine species, is remarkable and invites further study of the evolution and ecology of this bacterium.

**TABLE 1:** Genome features and ANI values for strains of Nitrosococcus oceani

Strain	Site of origin	Genome size (bp)	G+C content (%)	ANI value with NS58 (%)	GenBank accession no.	Reference(s) or source
ATCC 19707^T^	Atlantic Ocean (600 m depth)	3,522,111	50.32	99.9997	CP000127	1, 3
					CP000126	
AFC27	North Pacific	3,480,059	50.30	99.9952	ABSG00000000	1
C-27	Barbados Harbor	3,579,918	50.00	99.9978	JPGN00000000	1
AFC132	South Pacific	3,545,101	49.80	98.2816	JPFN00000000	1
NS58	Tokyo Bay (coastal sediment)	3,508,376	50.33	100	BJWX00000000	This study
					AP019848	

### Data availability.

The raw reads have been deposited in the DDBJ Sequence Read Archive (DRA) under the accession number DRA008708. This whole-genome shotgun sequencing project has been deposited in DDBJ/ENA/GenBank under the accession numbers BJWX00000000 (53 contigs) and AP019848 (plasmid pNS58).
